# A flexible ChIP-sequencing simulation toolkit

**DOI:** 10.1186/s12859-021-04097-5

**Published:** 2021-04-20

**Authors:** An Zheng, Michael Lamkin, Yutong Qiu, Kevin Ren, Alon Goren, Melissa Gymrek

**Affiliations:** 1 Department of Computer Science and Engineering, University of California San Diego, 9500 Gilman Drive, La Jolla, CA 92093 USA; 2 Department of Bioengineering, University of California San Diego, 9500 Gilman Drive, La Jolla, CA 92093 USA; 3 School of Computer Science, Carnegie Mellon University, 5000 Forbes Avenue, Pittsburgh, PA 15213 USA; 4 Department of Mathematics, Massachusetts Institute of Technology, 77 Massachusetts Avenue, Cambridge, MA 02139 USA; 5 Department of Medicine, University of California San Diego, 9500 Gilman Drive, La Jolla, CA 92093 USA

**Keywords:** Bioinformatics, Epigenomics, ChIP-sequencing, Simulation tool, Command-line program

## Abstract

**Background:**

A major challenge in evaluating quantitative ChIP-seq analyses, such as peak calling and differential binding, is a lack of reliable ground truth data. Accurate simulation of ChIP-seq data can mitigate this challenge, but existing frameworks are either too cumbersome to apply genome-wide or unable to model a number of important experimental conditions in ChIP-seq.

**Results:**

We present ChIPs, a toolkit for rapidly simulating ChIP-seq data using statistical models of key experimental steps. We demonstrate how ChIPs can be used for a range of applications, including benchmarking analysis tools and evaluating the impact of various experimental parameters. ChIPs is implemented as a standalone command-line program written in C++ and is available from https://github.com/gymreklab/chips.

**Conclusions:**

ChIPs is an efficient ChIP-seq simulation framework that generates realistic datasets over a flexible range of experimental conditions. It can serve as an important component in various ChIP-seq analyses where ground truth data are needed.

**Supplementary Information:**

The online version contains supplementary material available at 10.1186/s12859-021-04097-5.

## Background

Chromatin immunoprecipitation followed by next generation sequencing (ChIP-seq) is a widely used technology for genome-wide mapping of the location of histone modifications (HMs) or DNA-associated proteins such as transcription factors (TFs) and chromatin regulators (CRs) [1]. Dozens of methods have been developed for quantitatively analyzing ChIP-seq data, including peak callers [2, 3] and differential binding tools [4, 5]. A major challenge in training and evaluating these methods as well as interpreting their results is a lack of reliable ground truth data: in most cases, the actual locations and strengths of binding sites or regions enriched for certain histone modifications are not known and cannot be reliably measured using orthogonal experimental techniques. Computational analysis of ChIP-seq is further complicated by multiple sources of noise introduced during the experimental process, including inefficiency or non-specificity of antibodies, PCR artifacts, and sequencing errors [6, 7].

Accurate simulation of ChIP-seq data can mitigate this challenge, but existing frameworks [8–11] are either cumbersome to apply genome-wide or do not accurately capture important sources of variation present in real data such as pulldown non-specificity, fragment length variability, or sequencing errors (Additional file 1: Supplementary Table 1). Importantly, existing simulation tools are not capable of inferring model parameters from real ChIP-seq datasets, making it difficult to choose realistic simulation settings.

Here, we present ChIPs (ChIP-seq simulator), a flexible toolkit for rapidly simulating ChIP-seq data based on realistic statistical models. ChIPs is a computationally efficient command-line solution that allows users to easily specify a wide range of parameters modeling key experimental steps and to infer these parameters from existing datasets. We demonstrate the applicability of ChIPs for evaluating the impact of various experimental conditions and for benchmarking computational analysis tools.

## Implementation

### Framework architecture

**Fig. 1:**
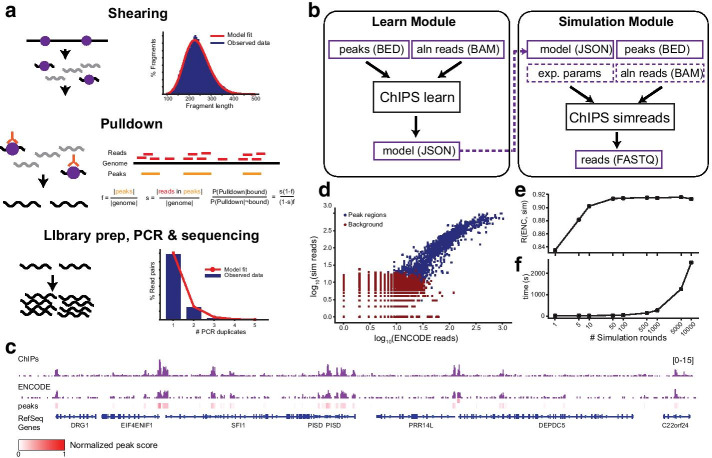
ChIPs overview. **a** Overview of the ChIPs model. ChIPs models four steps: shearing (top), pulldown (middle), PCR (bottom), and sequencing. Top: the dark blue histogram shows an example fragment length distribution from real paired end ChIP-seq data. The red line shows the best fit gamma distribution. Middle: pulldown is modeled using two parameters; *f* (the fraction of the genome bound by the factor) and *s* (the probability that a pulled down fragment is bound. Bottom: The dark blue histogram shows an example of a distribution of the numbers of PCR duplicates in real ChIP-seq data. The red line shows the best fit geometric distribution. **b** Schematic of ChIPs modules. The learn module takes an existing ChIP-seq experiment (aligned reads and peaks) and learns model parameters (see Additional file 1: Supplementary Table 2). The simulation module takes as input a set of peaks and model parameters, simulates a ChIP-seq experiment, and returns raw reads in FASTQ format. Model parameters input to the simulation module may either be learned from an existing ChIP-seq dataset (dashed arrow) or manually specified to capture planned experimental conditions. Purple borders represent input or output files and black boxes denote ChIPs commands. Boxes with solid lines denote required inputs. Boxes with dashed borders denote optional inputs. “Exp. params” denotes experimental parameters including the number of reads, read length, and number of simulation rounds. “Aln reads” denotes aligned reads in BAM format. **c** Example coverage profiles of real versus simulated data. The bottom track shows peaks identified by ENCODE, with normalized peak scores between 0 to 1 colored based on a gradient from white to red. The middle track shows coverage profiles based on aligned reads from ENCODE, and the top track shows coverage profiles based on ChIPs simulations. Coverage profiles were generated using IGV. Coverage profiles may also be viewed interactively at https://tinyurl.com/y7x6ggdq. **d** Concordance of read counts between simulated versus real ChIP-seq data. chr22 was divided into non-overlapping 5 kb bins. The scatter plot shows the comparison of read counts per bin for bins overlapping peaks (dark blue) or background regions (dark red). The x- and y-axes are on a log10 scale. The plot shown is for 100 simulated genome copies. **e** Read count correlation between real and simulated data as a function of number of simulated genome copies. For each number of copies, the correlation was computed between read counts in 5 kb bins overlapping input peaks. The x-axis is on a log10 scale. **f** Simulation run time as a function of number of simulated genome copies. The x-axis is on a log10 scale

ChIPs models each major ChIP-seq step (shearing, immunoprecipitation, pulldown, PCR, and sequencing) as a distinct module (Fig. 1a). It assumes binding sites for the target epitope and their binding scores (probabilities) are known. Notably, for histone modifications, we use binding to refer to genomic localization with the target modification, although the DNA itself is not typically bound by the modification. Importantly, each step is modeled in a way that key parameters can be inferred from existing datasets.

*Step 1: Shearing* Cross-linked DNA is first sheared to a target fragment length, for instance by sonication or enzymatic approaches [12]. ChIPs models fragment lengths using a gamma distribution (Fig. 1a; top) based on empirical observation of fragment distributions which have long right tails. The fragment length distribution parameters are either trivially inferred from paired end read alignments or are approximated from single end data using a heuristic method (Additional file 1: Supplementary Methods, Supplementary Figure 1).

*Step 2: Immunoprecipitation* Sheared cross-linked DNA is subject to immunoprecipitation, during which an antibody is used to enrich the pool of fragments for those bound to the epitope of interest. To model this imperfect process, we quantify the ratio, $\alpha$, of the probability of pulling down a bound versus unbound fragment. This modeled ratio is specific to each ChIP-seq experiment and depends on the antibody specificity as well as the fraction of the genome bound by the factor of interest. Let *f* be the fraction of the genome bound by the factor of interest and *s* be the fraction of pulled down reads that originate from true binding sites. We can approximate $\alpha$ using Eq. 1. A detailed derivation of this ratio is provided in Additional file 1: Supplementary Methods.

1 $$\begin{aligned} \alpha = \frac{s(1-f)}{(1-s)f} \end{aligned}$$

The parameters *f* and *s* can be directly inferred from real data based on binding sites or enriched regions (peaks) identified by various peak-calling methods (Additional file 1: Supplementary Methods, Supplementary Figure 2).

*Step 3: PCR* PCR is used to amplify pulled down fragments before sequencing. Let $n_i$ represent the number of reads (or read pairs) with *i* PCR duplicates (including the original fragment). $n_i$ is modeled using a geometric distribution, where *p* gives the probability that a fragment has no PCR duplicates. The parameter *p* is estimated as $1/\overline{n}$, where $\overline{n} = \frac{\sum _{i=1}^\infty (in_i)}{\sum _{i=1}^\infty n_i}$.

*Step 4: Sequencing* Finally, amplified fragments are subject to either paired end or single end sequencing. Sequences are based on an input reference genome using the coordinates of each fragment. We model the per-base pair substitution, insertion, and deletion rates (Additional file 1: Supplementary Table 2).

### Implementation details

ChIPs is implemented as an open source C++ project with source code publicly available on Github: https://github.com/gymreklab/chips. It consists of two utilities: simreads and learn (Fig. 1b). The simreads module takes in ChIP-seq model parameters and experimental settings (Additional file 1: Supplementary Table 2), and outputs simulated reads. Input parameters can either be set by the user to mimic a future ChIP-seq experiment or learned from existing data using the learn module. The user must additionally specify the number of simulation rounds, which denotes the number of times the input reference genome is processed by ChIPs. Notably, this number is related, but not directly comparable, to the number of experimentally processed cells, since pulldown efficiency is not directly included in our current model. We have found that in most settings 25–100 and 1000 rounds work well for HMs and TFs, respectively. Full implementation details and methods for benchmarking experiments are provided in Additional file 1: Supplementary Methods.

## Results

### Comparison of ChIPs simulation results to real ChIP-seq data

We evaluated ChIPs using ChIP-seq data generated by the ENCODE Project [13] for an example histone modification H3K27ac in the GM12878 cell line. To evaluate the effect of varying the number of simulation rounds, we simulated reads on chromosome 22 using parameters inferred from real data over a range of simulation rounds (1–10,000). Run time for chromosome 22 ranged from 11 s (1 round) to 15 min (10,000 rounds). Resulting reads were aligned to the hg19 reference genome using BWA-MEM [14], and duplicates were flagged using Picard [15]. Visual inspection of the resulting coverage profiles shows high similarity between real and simulated data (Fig. 1c).

Next, we compared read counts in bins of 5kb and found high correlation between real and simulated data in bins containing at least one peak (Fig. 1d; Pearson $r=0.91$; $p<10^{-200}$; n=1,232 bins; 100 simulation rounds). Further, correlation with ENCODE data increased as a function of the number of simulation rounds but plateaued around 100, suggesting little gain in simulating additional rounds compared to the time tradeoff (Fig. 1e–f). We repeated this analysis on multiple additional HMs and TFs in GM12878 with similar results (Additional file 1: Supplementary Figure 3).

### Benchmarking against existing ChIP-seq simulators

We next benchmarked ChIPs against existing ChIP-seq simulators, which are summarized in Additional file 1: Supplementary Table 1. We focused on two recent methods: (1) ChIPulate [9] is a method for simulating TF ChIP-seq data using detailed modeling of locus-specific binding energies. ChIPulate only simulates reads at bound regions, and does not simulate background fragments outside of peak regions, a key feature of real ChIP-seq datasets related to the antibody specificity. (2) isChIP [11] is a command-line method for simulating ChIP-seq data based on a set of input peaks, model parameters, and sequencing parameters. While isChIP performs a similar task to ChIPs, it is not able to infer model parameters from existing datasets, which is a key feature of ChIPs. A more detailed description of model differences between these tools is provided in  Additional file 1: Supplementary Note.

We used ChIPs, ChIPulate, and isChIP to simulate ChIP-seq data based on six different ENCODE datasets including 3 HMs (H3K4me1, H3K4me3, and H3K27ac) and 3 TFs (BCLAF1, IKZF1, and NFYA) (Additional file 1: Supplementary Table 3). For each dataset, we used the three methods to simulate data for chr22 based on ENCODE peaks and with settings meant to capture similar properties of the ENCODE data, including read length and read number. We additionally inferred model parameters using our learn module and used these models to set appropriate simulation options for each tool when possible (Additional file 1: Supplementary Methods). For each tool, we varied the number of simulation rounds (similar to the number of cells) from 1 to 10,000. ChIPulate simulations took approximately 80 min to complete regardless of the number of simulation rounds, although subsequent simulations reused intermediate files and were faster. isChIP consistently achieved the fastest run time (e.g. 0.8 min for 1000 rounds on H3K27ac compared to 4.9 min for ChIPs). For both isChIP and ChIPs, simulation time was far less than the run time of downstream steps of sequence alignment and peak calling.

For each simulated dataset, we compared to real data using two methods. First, similar to above, we aligned simulated reads to the hg19 reference genome and compared read counts in 1kb bins containing at least one peak. As expected, correlation with ENCODE increases for all tools with additional simulation rounds (Additional file 1: Supplementary Figure 4a). In all evaluated conditions we found that ChIPs showed superior correlation with ENCODE data. ChIPs perforomance was virtually unchanged when using models based on paired versus single end data (Additional file 1: Supplementary Figure 4a).

Second, to evaluate how well each tool captures noise in real data, we examined the distribution of read counts in bins with and without peaks (referred to as peak and background regions, Additional file 1: Supplementary Figure 4b) between simulated and real data. We further visualized these trends using simulated coverage profiles and ENCODE data using the Integrative Genomics Viewer [16] (Additional file 1: Supplementary Figure 5). In all cases, data simulated by ChIPs most closely matches read count distributions in peak versus background regions in the ENCODE data. As expected, almost no reads from ChIPulate align to background regions. For isChIP, we found that using the default background noise level resulted in far higher signal to noise ratios than in the real data. We attempted to more closely match ENCODE data by performing an additional experiment with increased background noise. This in some cases alleviated the bias but still matched less closely than ChIPs data (Additional file 1: Supplementary Figure 4b).

Taken together, our benchmarking results show that ChIPs most accurately captures properties of real ChIP-seq data. Further, whereas ChIPs could learn appropriate model parameters from existing datasets, the alternative tools first required detailed user involvement to determine realistic simulation settings for a particular dataset type. While we cannot rule out that further tuning of parameters for each method could achieve higher correlation, we found that without a method to infer parameters from existing data that it was difficult to choose optimal simulation settings.

### Demonstration of ChIPs applications

**Fig. 2:**
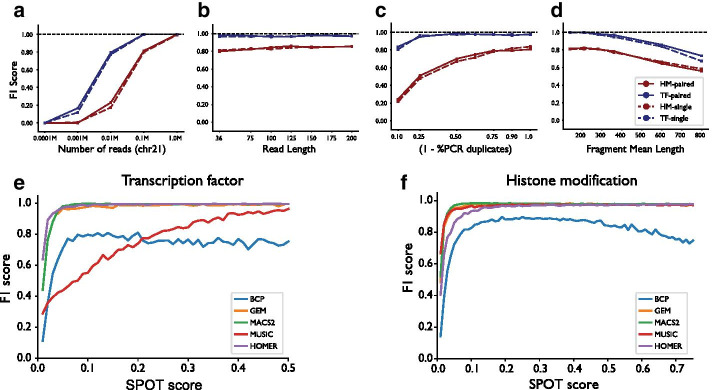
Example ChIPs applications. **a**–**d** Evaluation of the effects of varying experimental parameters on peak calling performance. Results are based on simulation of generic TF and HM datasets for chr21 as described in Additional file 1: Supplementary Methods. In each plot the y-axis shows the F1 score computed by comparing ground truth peaks to those inferred from simulated datasets using MACS2. **a** F1 score as a function of the total number of reads simulated from chr21. **b** F1 score as a function of read length. **c** F1 score as a function of PCR duplicates. The x-axis gives the parameter *p*, which can be interpreted as the percent of fragments with no PCR duplicates (Additional file 1: Supplementary Table 2). **d** F1 score as a function of mean fragment length (bp). Red = HM; Blue = TF; solid lines=paired end reads; dashed lines=single end reads. **e**–**f** Evaluation of various peak calling methods on simulated TF (**e**) and HM (**f**) datasets with different noise levels. Noise levels are quantified using *s*, the fraction of pulled down reads that originate from true binding sites. Blue = BCP; orange = GEM; green = MACS2; red = MUSIC; purple = HOMER

We next used ChIPs to evaluate the effects of varying experimental parameters on the ability to accurately detect TF or HM peaks. We examined read number, read length, PCR duplicate rate, and antibody specificity, and used ChIPs to simulate a series of datasets by varying each parameter. We generated two sets of simulated data to capture general properties such as peak size distributions characteristic of HMs or TFs (Supplementary Methods). We used MACS2 [2] to call peaks on the resulting datasets after alignment and duplicate marking. Each simulated dataset was evaluated based on the fraction of peaks recovered by the simulated datasets (recall), the fraction of called peaks that were correct (precision), and the combination of precision and recall (F1 score) (Fig. 2a–d, Additional file 1: Supplementary Figure 6). Simulated datasets recapitulate expected trends. Peak calling accuracy increases most dramatically as a function of the total number of reads, and performance decreases for datasets with larger fragment lengths or higher rates of PCR duplicates. Read length and the choice of single versus paired end reads have little impact on peak calling performance in mappable regions of the genome included in our analysis.

Finally, to demonstrate the ability of ChIPs to generate ground truth data for evaluating analysis tools, we compared performance of multiple peak calling methods on simulated datasets. We focused on five representative tools: MACS2 [2], GEM [17], MUSIC [3], BCP [18], and HOMER [19]. We measured peak calling performance using simulated datasets representative of generic HMs or TFs as described above but with varying degrees of non-specific binding (ChIPs *s* parameter, commonly referred to as a SPOT or FRIP score [7]; Fig. 2e–f, Additional file 1: Supplementary Figure 7). As expected, in all settings peak calling performance increased as a function of *s*. No method acheived superior performance across all datasets or metrics. For TFs, GEM, MACS2, and HOMER showed similarly high F1 scores for datasets with $s>0.05$. For HMs, all tools except BCP showed high F1 scores across a range of *s* values. Notably, our analysis captures only a small subset of possible dataset parameters, and it is likely that results will vary depending on specific datasets. Previous work has performed an extensive evaluation of various peak calling methods [20].

## Conclusions

In summary, we present ChIPs, an efficient command-line program that can rapidly generate realistic ChIP-seq data over a wide range of experimental conditions. ChIPs can infer model parameters from real data and generate simulated data for both TF and HMs. The whole process takes just seconds to minutes for most applications. Our framework is modular, allowing future integration of alternative or improved models at various simulation steps. For example, we can further model multiple types of biases, such as the ones introduced by specific cross-linking steps. Or we can model the biases introduced during pulldown by inherent factors such as GC content or DNA accessibility.

In this study, we benchmarked ChIPs against existing simulation tools and compared simulation results with a broad range of real ChIP-seq datasets as ground-truth. While all these tools could model multiple aspects of ChIP-seq data, we found that ChIPs most closely captures the properties of real ChIP-seq datasets. Another advantage of ChIPs is that, among all simulation tools benchmarked in this study, ChIPs is the only method capable of inferring model parameters from real data, allowing realistic simulation.

We demonstrated the utility of ChIPs in several usage scenarios, including benchmarking peak calling methods and measuring the effects of experimental conditions on peak detection. Some potential future applications include (1) evaluating the effects of genetic variation, such as SNPs, indels, or repeats, on observed ChIP-seq signals, (2) modeling effects of biological processes, such as DNA replication, on ChIP-seq signals, and (3) analyzing effects of spike-in normalization controls. Overall, we envision our framework will serve as a valuable resource for future efforts in ChIP-seq analysis.

## Availability and requirements

- Project name: ChIP-seq simulator.
- Project home page: https://github.com/gymreklab/chips.
- Operating system: CentOS Linux release 7.8.2003 (Core), macOS Catalina v10.15.7.
- Programming language: C++.
- Other requirements: gcc 4.9.2 or higher.
- License: GNU General Public License v3.0.
- Any restrictions to use by non-academics: None.

## Supplementary information

**Additional file 1:** Supplementary Methods, Supplementary Note, Supplementary Figures and Tables.

## Data Availability

The data that support the findings of this study are publicly available from the ENCODE Project [https://www.encodeproject.org/files/]. The accession ID for each dataset can be found in Additional file 1: Supplementary Table 3. The ChIPs source code, installation steps, and usage instructions are available at https://github.com/gymreklab/chips.

## Abbreviations

ChIP-seq: Chromatin immunoprecipitation sequencing
TF: Transcription factor
HM: Histone modification
CR: Chromatin regulators
ChIPs: ChIP-seq simulator
PCR: Polymerase chain reaction
ENCODE: Encyclopedia of DNA Elements
