# Supplementary material for: A flexible ChIP-sequencing simulation toolkit
Source: BMC Bioinformatics. 2021 Apr 20;22:201. doi: 10.1186/s12859-021-04097-5 (PMC8056602; doi:10.1186/s12859-021-04097-5)
Supplement: Supplementary file 1 — Additional file 1: Supplementary Methods, Supplementary Note, Supplementary Figures and Tables. [file 12859_2021_4097_MOESM1_ESM.pdf]

Supplementary Material - Zheng, *et al.*

1 Supplementary Methods

1.1 Model details

This section provides additional details on the ChIPs model.

Step 1: shearing

In the case of paired end reads, fragment lengths can be determined trivially from the mapping locations of paired reads. The observed fragment length (X_i) for each read pair i can be computed based on the mapping coordinates of the two reads. The learn module randomly selects 5,000 uniquely aligned read pairs from the input BAM for fitting a gamma distribution. Read pairs are filtered to remove fragments that are unaligned, not properly paired, marked as duplicates or marked as secondary alignments. Read pairs are further filtered to remove fragments with length greater than 3 times the median length of selected fragments. The mean fragment length is easily computed as $\mu = \frac{\sum_{i=1}^{n} X_i}{n}$, where n is the number of fragments remaining after filtering. We then use the method of moments to find maximum likelihood estimates of the gamma distribution shape (k) and scale parameters (θ):

$$\theta = \frac{1}{n\mu}\sum_{i=1}^{n}(X_i - \mu)^2 \tag{1}$$

$$k = \frac{\mu}{\theta} \tag{2}$$

For single end reads, individual fragment lengths are not directly observed. We outline an approximate method for estimating fragment length distributions from single end data in the section "Inferring fragment lengths from single end reads" below.

Step 2: immunoprecipitation

We use B_i to denote that fragment i is bound, $\overline{B_i}$ to denote that fragment i is unbound, and D_i to denote that fragment i is pulled down. For fragment i, assuming the probability that a given bound fragment is pulled down is approximately constant over all fragments, the probability of being pulled down can then be written as:

$$\begin{aligned} P(D_i) &= P(D, B_i) + P(D, \overline{B_i}) \\ &= P(D|B)P(B_i) + P(D|\overline{B})(1 - P(B_i)) \end{aligned}$$

where $P(D|B)$ denotes the probability that a given bound fragment will be pulled down and $P(D|\overline{B})$ denotes the probability that a given unbound fragment will be pulled down.

For any fragment i, we set the probability of it being bound $P(B_i)$ based on the scores of peaks it overlaps:

$$P(B_i) = \begin{cases} 0, & \text{if no overlap} \\ 1 - \Pi_{r \in R_i}(1 - S(r)), & \text{if overlap peak(s)} \end{cases} \tag{3}$$

where R_i is the set of all peak regions overlapping fragment i and $S(r)$ is the probability that peak r is bound. A method for estimating $S(r)$ is detailed below.

We compute conditional pulldown probabilities using Bayes' Rule:

$$P(D|B) = \frac{P(B|D)P(D)}{P(B)} \tag{4}$$

$$P(D|\overline{B}) = \frac{P(\overline{B}|D)P(D)}{P(\overline{B})} \tag{5}$$

where here $P(B|D)$ and $P(\overline{B}|D)$ represent averages across all fragments. We do not have a straightforward way to compute $P(D)$, the average probability that a fragment is pulled down, using only observed ChIP-seq reads since we do not actually observe fragments that are not pulled down. Thus, we do not attempt to compute these conditional probabilities directly. Instead, we take the ratio which cancels $P(D)$:

$$\alpha = \frac{P(D|B)}{P(D|\overline{B})} = \frac{P(B|D)P(\overline{B})}{P(\overline{B}|D)P(B)} \tag{6}$$

$P(B)$, or the probability that a fragment is bound on average, is equal to f, the fraction of the genome bound by the factor of interest. We can approximate f as the sum of the lengths of all peaks R weighted by their binding probabilities divided by the total length of the genome G:

$$f \approx \frac{1}{G} \sum_{r \in R} S(r) l_r \tag{7}$$

where G is the size of the reference genome, $S(r)$ is the probability peak region r is bound, and l_r is the length of peak r, assuming no overlap between peaks.

$P(B|D)$, or the probability that a fragment is bound given that it is pulled down, is a measure of the specificity of the antibody. Assuming the majority of reads falling in peaks are truly bound, we can roughly approximate this as the percent of fragments falling within peaks, which we denote as s.

Using these two metrics, f, and s (Supplementary Table 1), we can simplify the ratio α as:

$$\alpha = \frac{s(1-f)}{(1-s)f} \tag{8}$$

Since we only know the ratio α, for simplicity in simulations we set $P(D|B) = 1$ and $P(D|\overline{B}) = \frac{1}{\alpha}$. Substituting into the equation for $P(D_i)$ above, we compute the probability that fragment i is pulled down as:

$$P(D_i) = P(B_i) + \frac{1}{\alpha}(1 - P(B_i)) \tag{9}$$

where $P(B_i)$ is based on peak scores as defined above.

Note, in reality, $P(D|B)$ is likely to be much less than 1, since the pulldown process is inefficient and many fragments are lost. Further, the number is likely to vary largely across different experiments. Estimating the absolute value of $P(D|B)$ from real datasets is a topic of future work.

1.2 Inferring fragment lengths from single-end reads

To estimate the fragment length distribution from single-end reads, we assume the length distribution follows a gamma distribution with mean μ and variance v, and use reads located inside ChIP-seq peaks (provided as input) to estimate μ and v which are used to compute k and θ. This is a heuristic method that provides reasonable estimates of the fragment size distribution, which is sufficient for most applications.

For each peak $peak_i$, we keep track of two lists, $\{\text{start}\}_{\text{peak}_i}$ and $\{\text{end}\}_{\text{peak}_i}$. For each read overlapping peak_i, if the read is on the forward strand we add its start coordinate to $\{\text{start}\}_{\text{peak}_i}$. If the read is on the reverse strand we add its start coordinate to $\{\text{end}\}_{\text{peak}_i}$. The center point of this peak is calculated as:

$$\text{center}_{\text{peak}_i} = \frac{mean(\{\text{start}\}_{\text{peak}_i}) + mean(\{\text{end}\}_{\text{peak}_i})}{2} \tag{10}$$

For every peak_i we offset the coordinates in $\{\text{start}\}_{\text{peak}_i}$ and $\{\text{end}\}_{\text{peak}_i}$ by $\text{center}_{\text{peak}_i}$, so that the coordinates of start points and end points are symmetric around zero. We then concatenate lists from each peak to form $\{\text{start}\}$ and $\{\text{end}\}$:

$$\begin{aligned} \{\text{start}\} &= \oplus_{i=0}^{n}(\{\text{start}\}_{\text{peak}_i} - \text{center}_{\text{peak}_i}) \\ \{\text{end}\} &= \oplus_{i=0}^{n}(\{\text{end}\}_{\text{peak}_i} - \text{center}_{\text{peak}_i}) \end{aligned} \tag{11}$$

The mean fragment length μ can be estimated as:

$$\mu = mean(\{\text{end}\}) - mean(\{\text{start}\}) \tag{12}$$

We calculate the probability density functions, cumulative density functions and expected density functions for both $\{\text{start}\}$ and $\{\text{end}\}$. The expected density function $EDF(x)$ is defined as the expected deviation of a random element in the list to x:

$$\begin{aligned} EDF_{start}(x) &= E(|S - x|) \\ EDF_{end}(x) &= E(|E - x|) \end{aligned} \tag{13}$$

where S is a random element in $\{\text{start}\}$ and E is a random element in $\{\text{end}\}$.

After we compute μ, we can reduce the density function of the fragment length distribution from $p_{\mu,v}(x)$ to $p_v(x)$, the probability density function of the fragment length variance. We construct a score function $F(v)$ as shown below. Intuitively, if we have a correct guess of v, $F(v)$ should be equal to zero.

$$\begin{aligned} F(v) = E_v(|S + \tfrac{L}{2}|) + E_v(|E - \tfrac{L}{2}|) - E(|S + \tfrac{\mu}{2}|) - E(|E - \tfrac{\mu}{2}|) \\ E_v(|S + L/2|) = \textstyle\sum_{x=0}^{\infty} p_v(x) * EDF_{\text{start}}(-\tfrac{x}{2}) \\ E_v(|E - L/2|) = \textstyle\sum_{x=0}^{\infty} p_v(x) * EDF_{\text{end}}(\tfrac{x}{2}) \\ E(|S + \tfrac{\mu}{2}|) = EDF_{\text{start}}(x) \\ E(|E - \tfrac{\mu}{2}|) = EDF_{\text{end}}(x) \end{aligned} \tag{14}$$

where L represents a randomly chosen fragment length.

To find an optimal v that minimizes $|F(v)|$, we conduct a binary search between 1000 and 10,000.

In practice, we slightly offset the last two items in the score function in **Equation 14** to compute the score below, which results in slightly more accurate estimation of v on real data. This may be due to the fact that fragment length distributions are truncated on the left end, with little or no fragments with lengths less than 100bp observed, and thus do not follow a true gamma distribution.

$$F(v) = E_v\left(\left|S + \frac{L}{2}\right|\right) + E_v\left(\left|E - \frac{L}{2}\right|\right) - E\left(\left|S + \frac{\mu}{2} - \frac{E - \frac{\mu}{2}}{4}\right|\right) - E\left(\left|E - \frac{\mu}{2} - \frac{S + \frac{\mu}{2}}{4}\right|\right) \tag{15}$$

Examples of inferred fragment length distributions from single-end data compared to actual fragment length distributions for a variety of datasets is shown in Supplementary Figure 1.

1.3 ChIPs implemenetation details

Peak scores

A peak score $S(r)$ is defined for each input peak, where $S(r)$ gives the probability that a fragment overlapping the region is bound. Note we cannot directly estimate this probability from bulk ChIP-seq data, but assume variability in intensity across peaks is representative of different relative binding probabilities. Based on user input, ChIPs computes these binding probabilities either based on peak intensities given in an input BED file or based on read counts from an existing BAM file. If peak intensities are provided, $S(r)$ is computed as the score of peak r divided by the maximum peak intensity. If a BAM file is provided, $S(r)$ is defined as the number of reads overlapping peak r divided by the maximum number of reads overlapping any peak. In both cases, resulting scores $S(r)$ are between 0 and 1.

By specifiying the option --no-scale, users may directly input binding scores that will be treated directly as probabilities and will not be rescaled. Users may also specify --scale-outliers to remove peak intensities greater than 3 times the median score before rescaling peak intensities to reduce the effect of outlier peaks. In this case all peaks with scores greater than 3 times the median will be set to 1.

Learn implementation

The ChIPs learn module takes a set of input peaks (BED) and aligned reads (BAM) and learns parameters for (1) fragment length distribution (k, θ), (2) pull-down efficiency (f, s), and (3) PCR efficiency (p) (Supplementary Table 1). BAM files must have PCR duplicates marked using a tool such as Picard [1] to enable accurate estimation of PCR parameters. The learn module outputs model parameters to a JSON file that can be used as input to the simreads module. We found that the key pulldown parameter, α, learned, is relatively robust to the peak caller used to generate the input peak dataset (Supplementary Figure 2).

Simulation implementation

The simreads module takes in a set of peaks, model parameters (*e.g.*, from the learn module), experimental parameters (including read length, number of reads), and number of simulation rounds, and simulates raw sequencing reads in FASTQ format. First, each copy of the genome (equivalent to one simulation round) is randomly fragmented based on the specified fragment length gamma distribution. Next, we decide whether to pull down each fragment based on its overlap with input peaks based on $P(B_i)$ described above. Finally, we generate reads from the resulting pool of fragments based on input parameters (using the specified mode read length, number of reads, and mode [single/paired]). We output "PCR duplicates" of each sequenced read based on the input PCR rate p.

In practice, in each round the genome is processed in bins (default size 100kb) to avoid storing large fragment pools in memory. In a preprocessing step, we determine how many reads to generate from each simulation round based on the target number of output reads. ChIPs is parallelized by performing different simulation rounds on separate threads simultaneously.

1.4 Benchmarking experiments

Evaluating ChIPs performance

To evaluate ChIPs, we compared simulated reads to reads from real ChIP-seq experiments using read alignments and peaks generated by ENCODE [2]. All ENCODE accessions for reads and peaks are given in **Supplementary Table 3**.

We first ran the chips learn module to learn model parameters based on the ENCODE BAM and BED files for each TF or HM. We used learn parameters -c 7 -t bed --scale-outliers. The json model file output by the learn module was used as input to the simulate module. We performed various runs using an increasing number of simulation rounds (chips simulate argument --nc 1, 5, 10, 50, 100, 500, 1000, 5000, or 10000). We simulated single end reads for chr22 using the same read length as the corresponding ENCODE dataset and setting the number of reads to twice the number of aligned reads for chr22 in the real dataset since not all simulated reads will uniquely align back to the genome. Resulting reads were aligned to the hg19 reference genome using BWA-MEM [3] v0.7.12-r1039, and duplicates were flagged using Picard [1] v2.18.11.

We used the bedtools [4] (v2.27.1) makewindows command to generate a list of non-overlapping windows across chr22 and the bedtools multicov command to count the number of reads from both simulated or ENCODE BAM files falling in each 1kb window. We used the bedtools intersect command to determine the intersection of each bin with the input peak files. For each bin, we determined the Pearson correlation between log10 read counts in each simulated vs. ENCODE dataset after adding a pseudocount of 1 read to each bin.

Timing experiments were performed in a Linux environment running Centos 7.4.1708 on a server with 28 cores (Intel® Xeon® CPU E5-2660 v4 @ 2.00GHz) and 125 GB RAM using the UNIX ”time” command and are based on the ”sys” time reported.

Comparison to ChIPulate

We compared performance of ChIPulate [5] to ChIPs based on the datasets described in Supplementary Table 3 and shown in Supplementary Figures 5-6.

We first computed binding energies for each peak required as input. Binding probabilities $S(r)$ for each peak r were computed as described above by scaling peak scores based on ENCODE data to be between 0 and 1, similarly setting outlier peaks with scores higher than twice the median to have score 1. Then we computed binding energies for each region r as

$$E_r^{bound} = E_r^{unbound} + \texttt{chemical potential} + \ln[(1 - S(r))/S(r)] \tag{16}$$

$E_r^{unbound}$ and chemical potential are provided as command line inputs to ChIPulate. We set chemical potential to 0, which provided the best dynamic range across peak scores and highest correlation with real data. Background binding energy was set to the default (1.0).

ChIPulate parameters were set to achieve the same fragment length distributions, number of reads, and read lengths as in ENCODE data. Fragment length (--fragment-length) was set to the mean fragment size inferred by ChIPs learn. Read length (--read-length) was set to the read length of each dataset, ranging from 36bp to 101bp. Depth (-d) was set to the ratio of the number of reads in the ENCODE dataset mapped to chr22 divided by the number of peaks on chr22. We additionally set: p_amp=0.50, p_ext=0.54, --mu-A=0 based on examples shown in ChIPulate documentation. We used the parameter -n to vary the number of copies (simulation rounds) from 10 to 10,000. Runs with -n set to 1 or 5 failed and so were

excluded from analysis. All other parameters were set to default values. Read counts in windows of 1kb were compared between simulated and ENCODE datasets using bedtools multicov as described above.

Comparison to isChIP

We similarly evaluated performance of isChIP [6] v1.0 based on the datasets described in Supplementary Table 3 and shown in Supplementary Figures 4-5, setting parameters to mimic those of real datasets. Read length (-r) and the maximum number of reads (--rd-lim) were set based on read lengths and the number of reads aligned to chr22 in ENCODE data. The log of the mean fragment length (-L) was set based on the mean fragment length inferred by ChIPs learn. We provided ENCODE peaks as input with option --bscore 0. Using ENCODE peak scores directly (--bscore 7) resulted in very poor correlation with real data. We used the parameter --cells to vary the number of cells from 1 to 10,000.

Since we did not have a way to estimate foreground and background parameters (--ground option), we evaluated two settings. In the first, we set foreground to the spot score and background to the default value of 1. In the second, labeled "HighBG", we increased the background noise to 4.

Unless otherwise specified, we set the number of PCR cycles to 0 (--pcr 0). Attempts to run with even small numbers of PCR cycles resulted in unreliable output. Results from setting --pcr 10 are shown in Supplementary Figure 4.

Simulating benchmarking peaks

For experiments shown in **Figure 2**, we generated two sets of datasets meant to represent characteristics of either histone modification (HM) or transcription factor (TF) ChIP-seq datasets. We simulated a peak file for TF and HM each, with SP1 (bam=ENCFF001TYZ) and H3K27ac (bam=ENCFF411MHX) as templates. To generate peaks, we randomly placed peaks on the hg19 genome sampling peak lengths and scores from distributions observed on real data. In total, we simulated 29,579 non-overlapping peaks for the TF dataset and 77,413 for the HM dataset. Results in **Figure 2** are based on 350 TF peaks and 697 HM peaks on chr21.

Evaluating effects of experimental parameters

For evaluating experimental parameters (**Figure 2a-d**), we used ChIPs to simulate reads from chr21 based on either the HM or TF peak sets described above. Unless otherwise noted, all runs used ChIPs simulate parameters --scale-outliers --numcopies 100 --numreads 100000 --readlen 36 --gamma-frag 20,15 --pcr_rate 0.8. For TF datasets, unless otherwise noted, all runs used model options --spot 0.40 --frac 0.001. For HM datasets, unless otherwise noted, all runs used model options --spot 0.45 --frac 0.01. For evaluating read number, the --numreads option was set to 100, 1000, 10000, 100000, or 1000000. For evaluating read length, the --readlen option was set to either 36, 75, 100, 125, 150, or 200. For evaluating PCR rate, the --pcr_rate option was set to either 0.1, 0.25, 0.5, 0.625, 0.75, 0.875, or 1.0. For evaluating fragment length, the --gamma-frag option was set to either 10,14, 21,10, 40,7, 90,4, 200,3, or 400,2. In all cases separate datasets were generated for single and paired end reads using the --paired option to specify paired end output.

Resulting reads were aligned to the hg19 reference genome using BWA-MEM [3] v0.7.12-r1039, and duplicates were flagged using Picard [1] v2.18.11.

Evaluating peak callers

We conducted multiple sets of experiments simulating paired-end ChIP-seq datasets with different SPOT scores (s ranging 0.01-0.5 for TF data and 0.01-0.75 for HM data). For each simulated dataset we ran ChIPs simreads using the HM and TF model parameters described above and with additional options --paired --region chr21:1-48129895. We benchmarked peakcallers macs2 (v2.2.6), GEM (v3.4), MUSIC, BCP (peakranger v1.18), and HOMER. When running these peak callers, we applied their default parameter settings except flags indicating the types of input data (i.e. TF or HM).

To quantitatively evaluate the quality of the predicted peaks, we followed the metrics used in [7] and trimmed each peak to 200bp around its summit. Then, we calculated the number of real peaks that peak callers retrieved as well as the number of predicted peaks that overlap with real peaks.

2 Supplementary Note

Below we compare how key steps of the ChIP-seq process are modeled by ChIPs in comparison to other recently developed ChIP-seq simulators (ChIPulate [5] and isChIP [6]). These differences are also summarized in Supplementary Table 1.

Shearing

- **ChIPulate**: uses a fixed fragment length.
- **isChIP**: models variable fragment lengths drawn from a log-normal distribution with an optional size-selection step.
- **ChIPs**: models variable fragment lengths drawn from a gamma distribution. Gamma and log-normal distributions often appear highly similar, and in practice we have not found this difference in fragment length distributions to have a significant impact on simulation results.

Pulldown and binding efficiency

- **isChIP**: models the probability of loss of selected foreground vs. background fragments compared to generated fragments.
- **ChIPulate**: models binding efficiencies of each binding site, but does not consider background regions outside of specified binding sites.
- **ChIPs**: models pulldown efficiency using a Bayesian model based on the SPOT score (s) and fraction of the genome found (f), both of which can be inferred from real datasets and peaks generated by a variety of peak-calling algorithms (Supplementary Figure 2)

PCR

- **ChIPulate**: models the amplification efficiency of each region as well as the number of PCR cycles. Of the three tools, the ChIPulate PCR model is most advanced but also most computationally intensive.
- **isChIP**: copies each fragment 2^n times, where n is the number of PCR cyles. In practice, because the total number of fragments exponentially increases, this process explodes after several cycles. In our simulation experiments, we have found that using even small numbers of PCR cycles (e.g. 10) results in unreliable output.
- **ChIPs**: models the distribution of the number of duplicate reads. This parameter can be easily inferred from existing data and used to simulate realistic PCR duplicate patterns.

Sequencing

- **ChIPulate**: does not model sequencing errors
- **isChIP**: does not model sequencing errors
- **ChIPs**: models base substitution and indel rates based on real Illumina data.

3 Supplementary Figures

Supplementary Figure 1

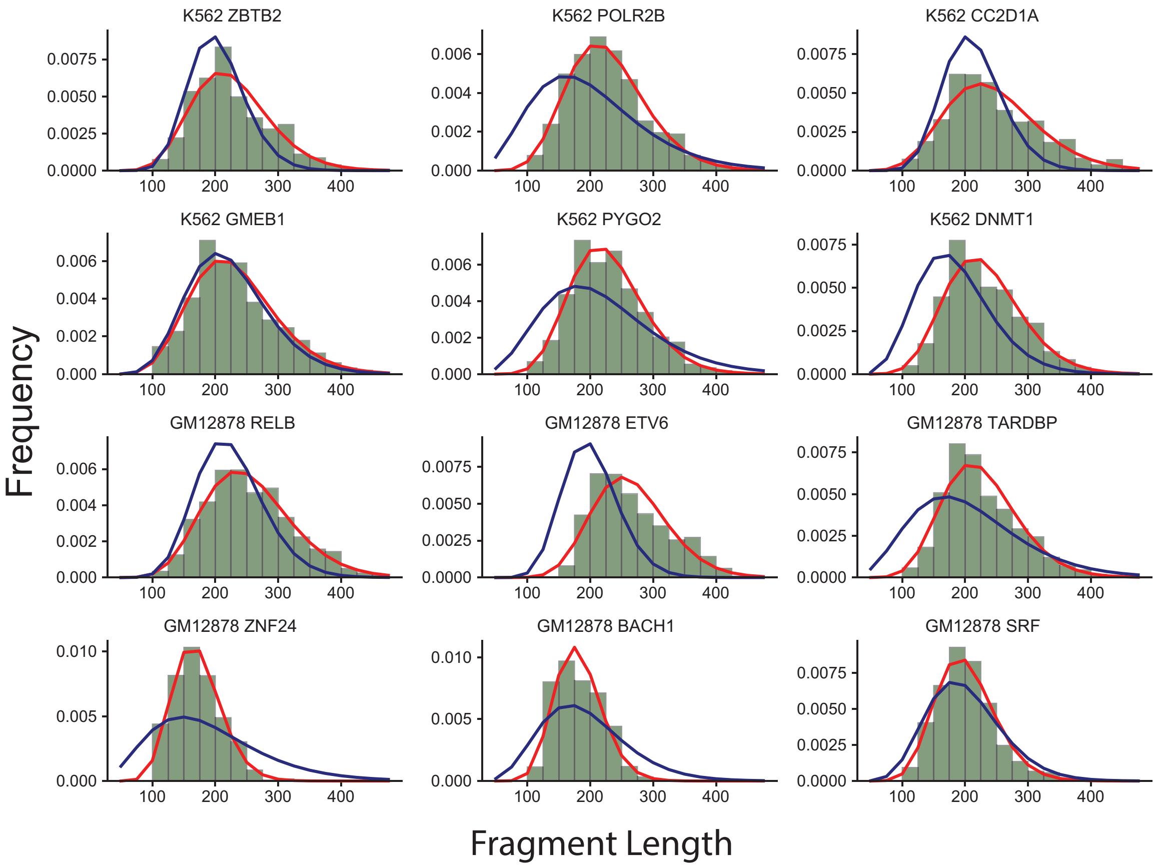

Inferring fragment length distributions from single-end reads. Green bars show a histogram of lengths of 10,000 randomly chosen fragments from GM12878 paired end ChIP-seq experiments. Red lines give the best fit gamma distribution learned using observed fragment lengths. Blue lines gives the fit inferred ignoring pair information using our novel method for learning fragment length distributions from single end data. ENCODE accessions are given in Supplementary Table 3.

Supplementary Figure 2

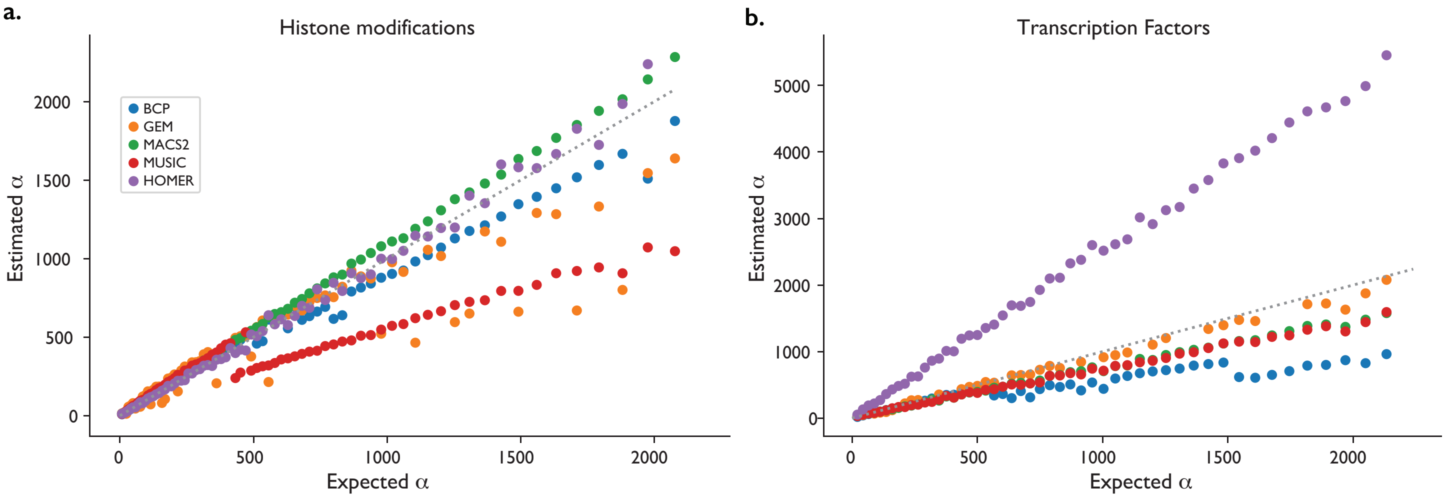

Evaluation of pulldown parameter estimation with input TF and HM peaks from different peak callers. Each dot in the plots indicates an individual experiment and shows the comparison between estimated and expected alpha. Alpha is the ratio of pulldown probabilities of bound regions to unbound regions as described in the Supplementary Methods. The expected alpha was used in simulating reads and the estimated alpha was computed using our ChIPs learn module. Blue=BCP; orange=GEM; green=MACS2; red=MUSIC; purple=HOMER. In a. (HM), α estimated based on MUSIC peaks becomes unreliable for simulated datasets with high s (SPOT) scores. This issue can be mitigated by applying tne "scale-outliers" option in the learn module which reduces the effect of outlier peaks. In b. (TF), the estimated alpha based on HOMER peaks is higher than expected. This is because HOMER is more stringent in deciding peak boundaries: the peak length from HOMER (mean=323.72bp) is smaller than the others (mean=746.22bp) and the ground truth (mean=448.80bp), resulting in the f value being underestimated and the α value being overestimated.

Supplementary Figure 3

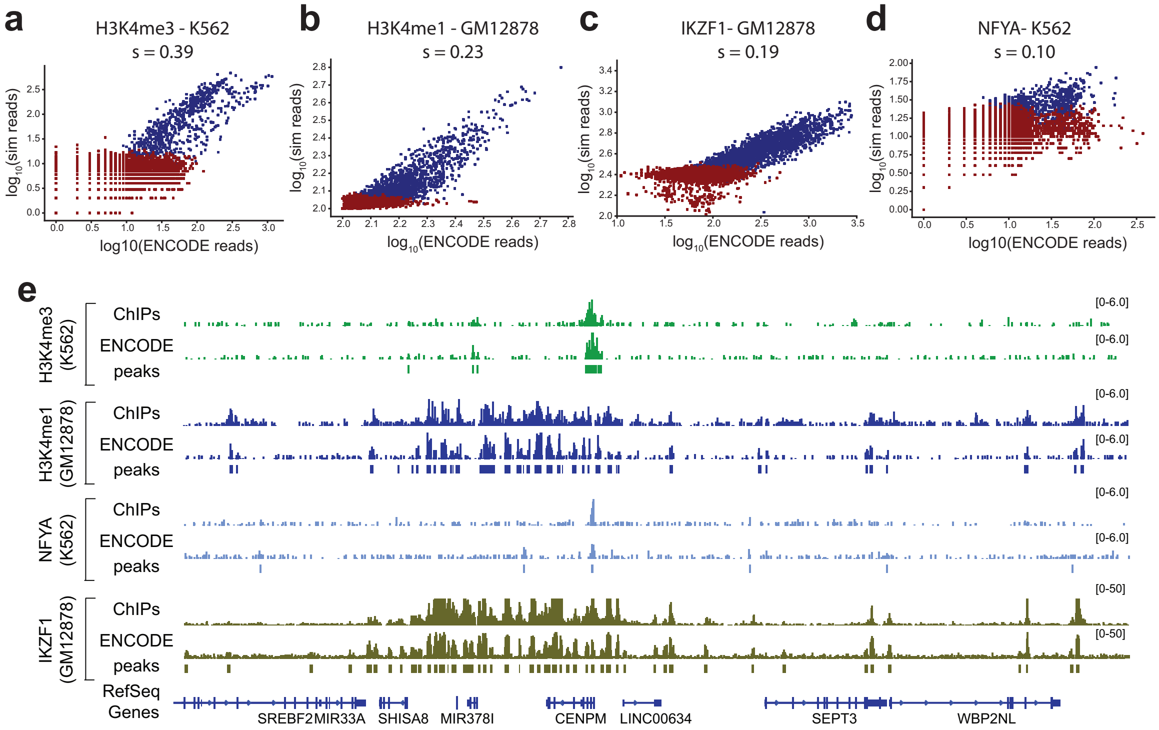

a-d Concordance of read counts between simulated vs. real ChIP-seq data. chr22 was divided into non-overlapping 5kb bins. The scatter plots show the comparison of read counts per bin for bins overlapping peaks (dark blue) or background regions (dark red). The x- and y-axes are on a log10 scale. The plot shown is for 100 simulated genome copies. e. Examples of coverage profiles of real vs. simulated data.

Supplementary Figure 4

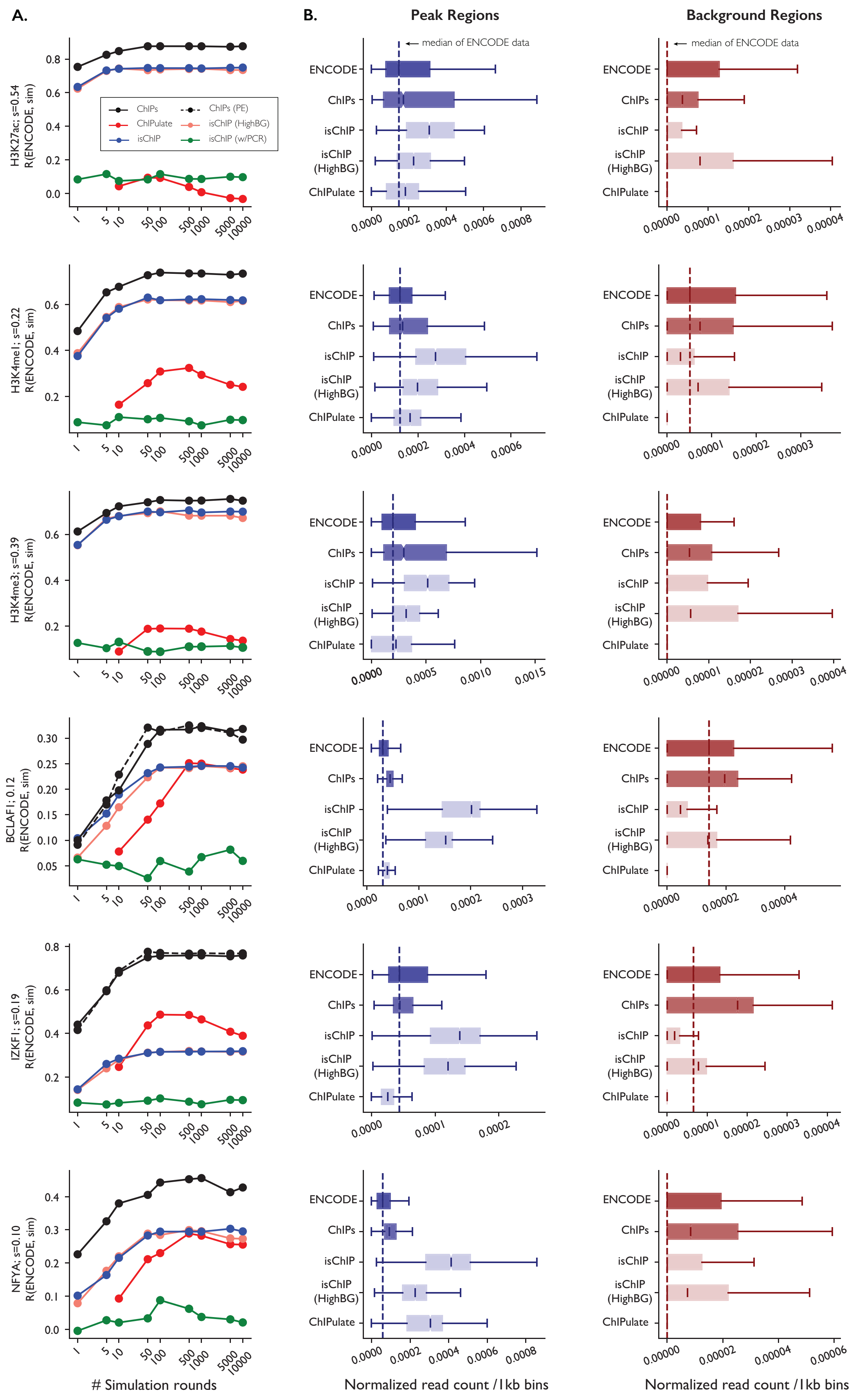

Benchmarking of ChIPs against existing simulators. Evaluation of ChIPs against existing methods was performed by comparing the simulation results of each to real ChIP-sequencing datasets covering a range of dataset types (both histone modifications and transcription factors), sequencing settings (single vs. paired end reads), and data qualities as measured by SPOT scores (s). a. The Pearson correlation between read counts of simulated vs. real (ENCODE) datasets for histone modifications and transcription factors was measured across a range of simulation rounds. isChIP was run with multiple parameter choices (isChIP: low background and no PCR, isChIP w/PCR: low background and 10 PCR cycles, isChIP HighBG: high background and no PCR). For datasets where paired-end data was available (BLCAF1 and IZKF1), we evaluated ChIPs using models learned from paired end data (dashed lines) and ignoring paired end information (solid lines). All other datasets are single-end. b. Box plots of the relative number of reads in peaks vs. background regions in simulated vs. real data per 1kb bins. Left (blue) are the peak regions, right (red) are the background regions. The median of the real (ENCODE) data is denoted as a dashed line in each plot. Read counts in each bin were normalized by the total number of reads aligned to chr22 for each dataset.

Supplementary Figure 5

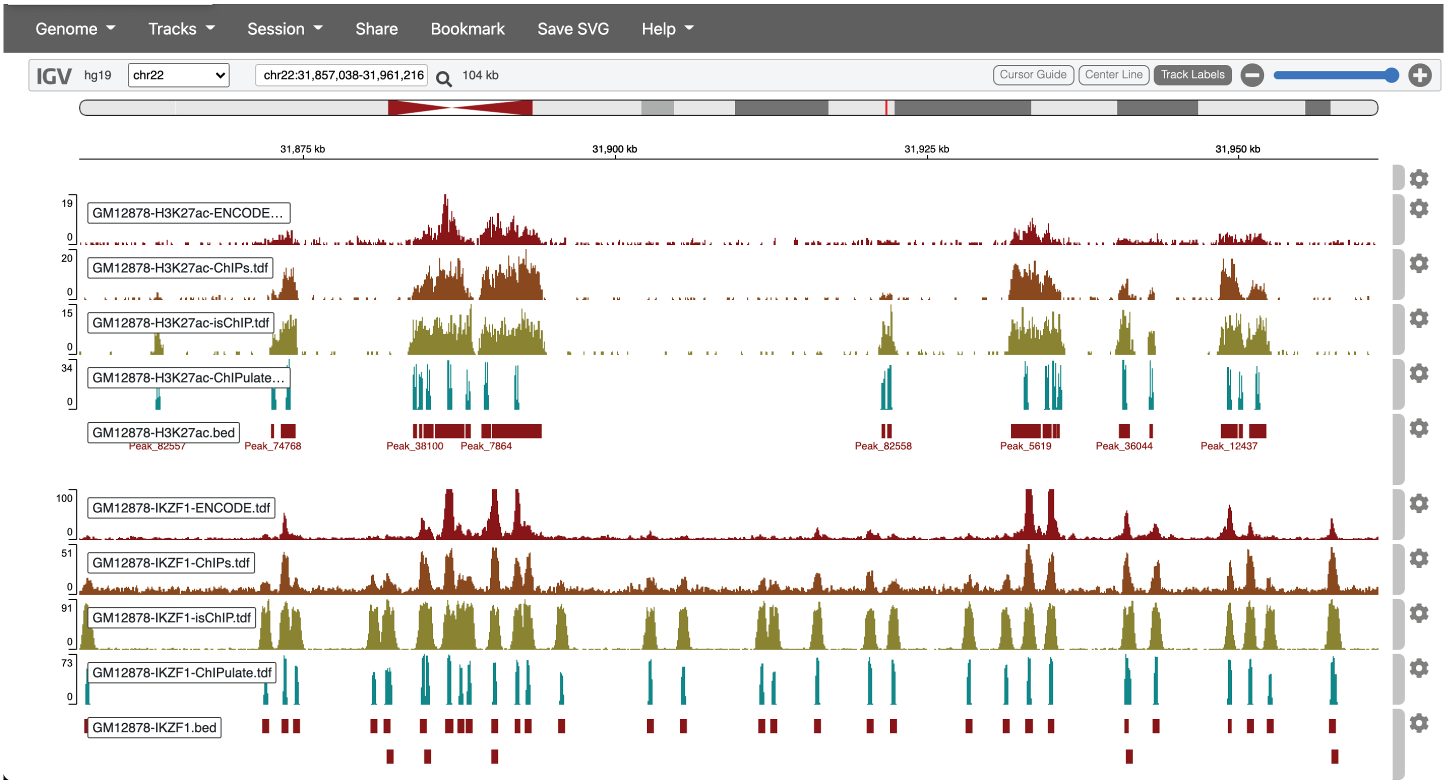

Visualization of coverage profiles for different ChIP-seq simulators Examples of coverage profiles of real vs. simulated data (brown=ENCODE, red=ChIPs, olive=isChIP, teal=ChIPulate. Brown bars show peaks called in the ENCODE data). Coverage profiles may also be viewed interactively at https://tinyurl.com/yblk3gb2

Supplementary Figure 6

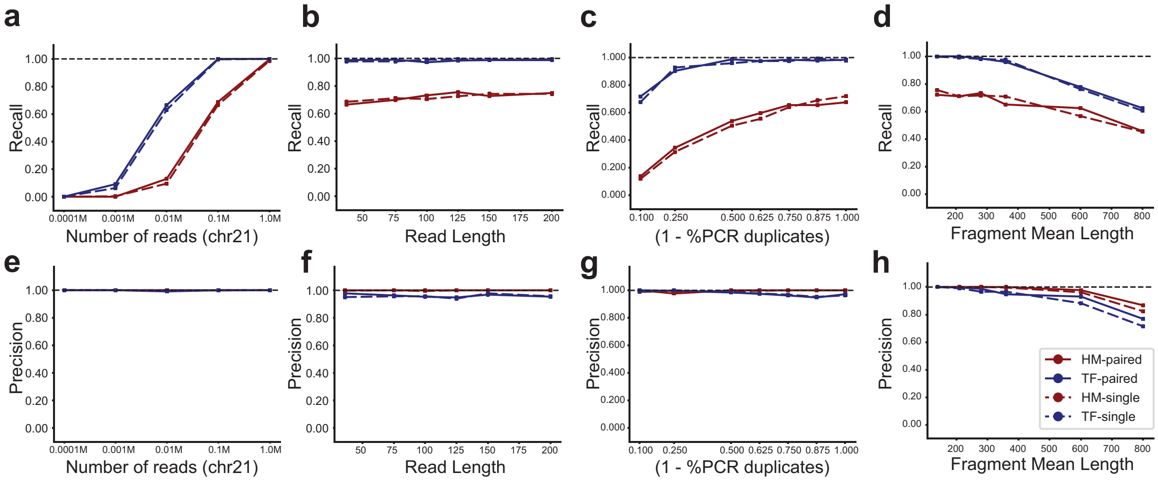

Evaluation of the effects of varying experimental parameters on peak calling performance. Results are based on simulation of generic TF and HM datasets for chr21 as described in the Supplementary Methods. For top plots the y-axis shows the recall (percent of real peaks inferred from simulated reads). For bottom plots the y-axis shows the precision (percent of inferred peaks that match simulated real peaks). a,e. Recall and precision as a function of the total number of reads simulated from chr21. b,f. Recall and precision as a function of read length. c,g. Recall and precision as a function of PCR rate. The x-axis gives the parameter p, which can be interpreted as the percent of fragments with no PCR duplicates (Supplementary Table 2). d,h. Recall and precision as a function of mean fragment length. Red=HM; Blue=TF; solid lines=paired end reads; dashed lines=single end reads.

Supplementary Figure 7

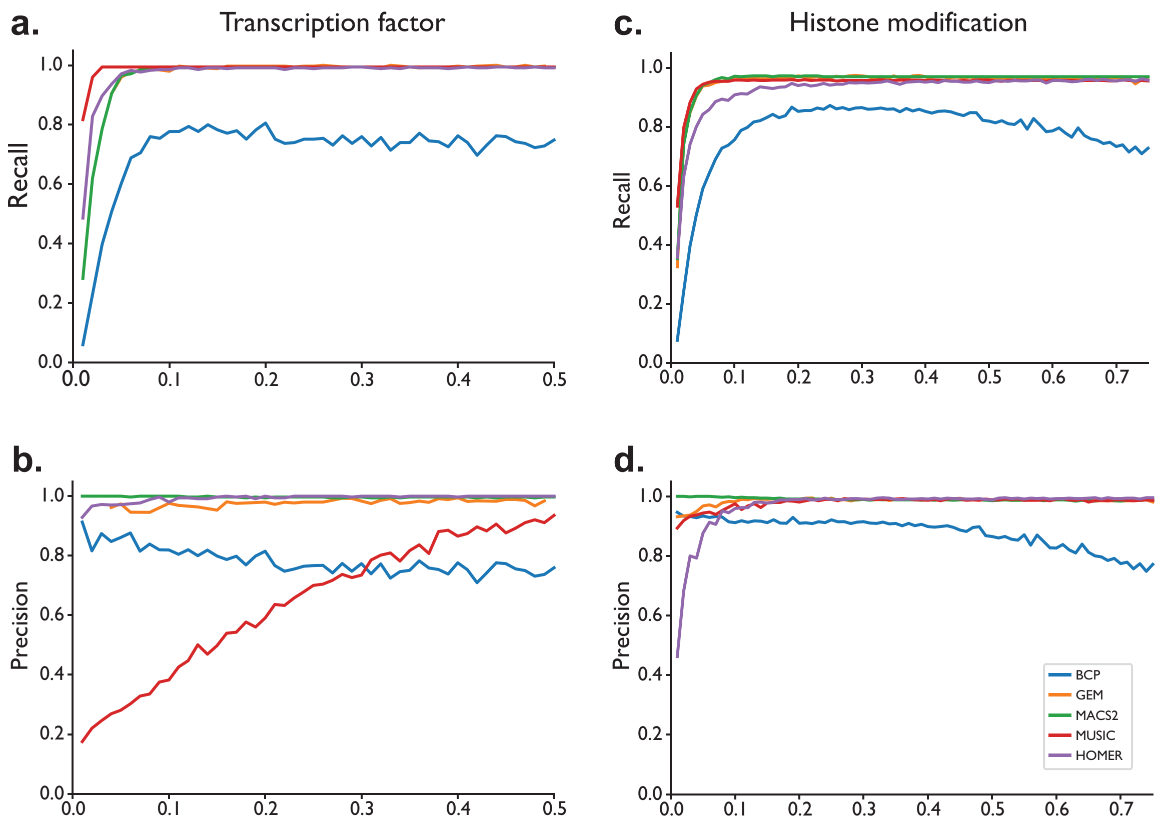

Evaluation of peak calling methods using simulated data. Noise levels are quantified using s, the fraction of pulled down reads that originate from true binding sites. Blue=BCP; orange=GEM; green=MACS2; red=MUSIC; purple=HOMER. Top plots show recall and bottom plots show precision.

4 Supplementary Tables

Supplementary Table 1

Feature	simchip [8]	ChIPulate [5]	isChIP [6]	ChIPs
Simulates background noise	Yes	No	Yes	Yes
Learns parameters from real data	No	No	No	Yes
Simulates peaks at user-specified regions	No	Yes	Yes	Yes
Simulates paired end reads	No	Yes	Yes	Yes
Simulates sequencing errors	Yes	No	No	Yes
Simulates PCR	No	Yes	Yes	Yes
Fragment length model	NA	fixed (1kb)	log-normal	gamma
User interface	R package	command-line	command-line	command-line
Approximate run time relative to ChIPs	-	16	0.16	1

Comparison of features in existing ChIP-seq simulation tools.

Supplementary Table 2: ChIPs parameters learned or input by users

**Parameters learned from real data (learn module)**			
Parameter	ChIP step	simreads argument	Description
k	Shearing	--gamma-frag float,float	Parameters of template lengths gamma distribution.
s	Pulldown	--spot float	SPOT score (percentage of reads falling in peaks).
f	Pulldown	--frac float	The fraction of the genome bound by the target factor.
p	PCR	--pcr_rate float	The percentage of fragments with no PCR duplicates.
**User-specified parameters**			
Parameter	ChIP step	simreads argument	Description
N	Input	--numcopies int	The number of rounds (genome copies) to simulate.
R	Sequencing	--numreads int	Target number of reads (or read pairs) to simulate.
l	Sequencing	--readlen int	Length of output reads.
	Sequencing	--paired	Whether to output paired vs. single end data.
e	Sequencing	--sub_rate float	Sequencing error rate.
d	Sequencing	--del_rate float	Sequencing deletion rate.
i	Sequencing	--ins_rate float	Sequencing insertion rate.

Supplementary Table 3: ENCODE accessions for benchmark datasets

Cell type	Epitope	BAM accession	PEAK accession	Result
K562	ZBTB2	ENCFF294LPO	ENCFF551YGS	Supplementary Figure 1
K562	POLR2B	ENCFF454PCU	ENCFF729LTY	Supplementary Figure 1
K562	CC2D1A	ENCFF054TBR	ENCFF051PEB	Supplementary Figure 1
K562	GMEB1	ENCFF809QWF	ENCFF154QJU	Supplementary Figure 1
K562	PYGO2	ENCFF288JVH	ENCFF078LXS	Supplementary Figure 1
K562	DNMT1	ENCFF987HMB	ENCFF958LLL	Supplementary Figure 1
GM12878	RELB	ENCFF708KIW	ENCFF355VTC	Supplementary Figure 1
GM12878	ETV6	ENCFF425VPI	ENCFF959JZX	Supplementary Figure 1
GM12878	TARDBP	ENCFF673WUM	ENCFF016QUV	Supplementary Figure 1
GM12878	ZNF24	ENCFF699QHD	ENCFF882PND	Supplementary Figure 1
GM12878	BACH1	ENCFF518TTP	ENCFF866OLZ	Supplementary Figure 1
GM12878	SRF	ENCFF387RFR	ENCFF500GHH	Supplementary Figure 1
GM12878	H3K27ac	ENCFF097SQI	ENCFF465WTH	Figure 1, Supplementary Figure 4
GM12878	IKZF1	ENCFF216YZE	ENCFF795PEX	Supplementary Figure 3,4
GM12878	H3K4me1	ENCFF252ZII	ENCFF966LMJ	Supplementary Figure 3,4
K562	H3K4me3	ENCFF681JQI	ENCFF127XXD	Supplementary Figure 3,4
K562	NFYA	ENCFF000YUR	ENCFF003WYE	Supplementary Figure 3,4
GM12878	BCLAF1	ENCFF671NSO	ENCFF222GJV	Supplementary Figure 4

BAM files and peak files corresponding to each accession can be found on the ENCODE Project [2] website: https://www.encodeproject.org/files/{accession}.

References

[1] B. Institute, "Picard tools." http://broadinstitute.github.io/picard/, 2018. Accessed: 2018-MM-DD; version X.Y.Z.

[2] I. Dunham, A. Kundaje, S. F. Aldred, P. J. Collins, C. A. Davis, F. Doyle, C. B. Epstein, S. Frietze, J. Harrow, R. Kaul, J. Khatun, B. R. Lajoie, S. G. Landt, B. K. Lee, F. Pauli, K. R. Rosenbloom, P. Sabo, A. Safi, A. Sanyal, N. Shoresh, J. M. Simon, L. Song, N. D. Trinklein, R. C. Altshuler, E. Birney, J. B. Brown, C. Cheng, S. Djebali, X. Dong, I. Dunham, J. Ernst, T. S. Furey, M. Gerstein, B. Giardine, M. Greven, R. C. Hardison, R. S. Harris, J. Herrero, M. M. Hoffman, S. Iyer, M. Kellis, J. Khatun, P. Kheradpour, A. Kundaje, T. Lassmann, Q. Li, X. Lin, G. K. Marinov, A. Merkel, A. Mortazavi, S. C. Parker, T. E. Reddy, J. Rozowsky, F. Schlesinger, R. E. Thurman, J. Wang, L. D. Ward, T. W. Whitfield, S. P. Wilder, W. Wu, H. S. Xi, K. Y. Yip, J. Zhuang, M. J. Pazin, R. F. Lowdon, L. A. Dillon, L. B. Adams, C. J. Kelly, J. Zhang, J. R. Wexler, E. D. Green, P. J. Good, E. A. Feingold, B. E. Bernstein, E. Birney, G. E. Crawford, J. Dekker, L. Elnitski, P. J. Farnham, M. Gerstein, M. C. Giddings, T. R. Gingeras, E. D. Green, R. Guig?, R. C. Hardison, T. J. Hubbard, M. Kellis, W. Kent, J. D. Lieb, E. H. Margulies, R. M. Myers, M. Snyder, J. A. Stamatoyannopoulos, S. A. Tenenbaum, Z. Weng, K. P. White, B. Wold, J. Khatun, Y. Yu, J. Wrobel, B. A. Risk, H. P. Gunawardena, H. C. Kuiper, C. W. Maier, L. Xie, X. Chen, M. C. Giddings, B. E. Bernstein, C. B. Epstein, N. Shoresh, J. Ernst, P. Kheradpour, T. S. Mikkelsen, S. Gillespie, A. Goren, O. Ram, X. Zhang, L. Wang, R. Issner, M. J. Coyne, T. Durham, M. Ku, T. Truong, L. D. Ward, R. C. Altshuler, M. L. Eaton, M. Kellis, S. Djebali, C. A. Davis, A. Merkel, A. Dobin, T. Lassmann, A. Mortazavi, A. Tanzer, J. Lagarde, W. Lin, F. Schlesinger, C. Xue, G. K. Marinov, J. Khatun, B. A. Williams, C. Zaleski, J. Rozowsky, M. R?der, F. Kokocinski, R. F. Abdelhamid, T. Alioto, I. Antoshechkin, M. T. Baer, P. Batut, I. Bell, K. Bell, S. Chakrabortty, X. Chen, J. Chrast, J. Curado, T. Derrien, J. Drenkow, E. Dumais, J. Dumais, R. Duttagupta, M. Fastuca, K. Fejes-Toth, P. Ferreira, S. Foissac, M. J. Fullwood, H. Gao, D. Gonzalez, A. Gordon, H. P. Gunawardena, C. Howald, S. Jha, R. Johnson, P. Kapranov, B. King, C. Kingswood, G. Li, O. J. Luo, E. Park, J. B. Preall, K. Presaud, P. Ribeca, B. A. Risk, D. Robyr, X. Ruan, M. Sammeth, K. S. Sandhu, L. Schaeffer, L. H. See, A. Shahab, J. Skancke, A. M. Suzuki, H. Takahashi, H. Tilgner, D. Trout, N. Walters, H. Wang, J. Wrobel, Y. Yu, Y. Hayashizaki, J. Harrow, M. Gerstein, T. J. Hubbard, A. Reymond, S. E. Antonarakis, G. J. Hannon, M. C. Giddings, Y. Ruan, B. Wold, P. Carninci, R. Guig?, T. R. Gingeras, K. R. Rosenbloom, C. A. Sloan, K. Learned, V. S. Malladi, M. C. Wong, G. P. Barber, M. S. Cline, T. R. Dreszer, S. G. Heitner, D. Karolchik, W. Kent, V. M. Kirkup, L. R. Meyer, J. C. Long, M. Maddren, B. J. Raney, T. S. Furey, L. Song, L. L. Grasfeder, P. G. Giresi, B. K. Lee, A. Battenhouse, N. C. Sheffield, J. M. Simon, K. A. Showers, A. Safi, D. London, A. A. Bhinge, C. Shestak, M. R. Schaner, S. K. Kim, Z. Z. Zhang, P. A. Mieczkowski, J. O. Mieczkowska, Z. Liu, R. M. McDaniell, Y. Ni, N. U. Rashid, M. J. Kim, S. Adar, Z. Zhang, T. Wang, D. Winter, D. Keefe, E. Birney, V. R. Iyer, J. D. Lieb, G. E. Crawford, G. Li, K. S. Sandhu, M. Zheng, P. Wang, O. J. Luo, A. Shahab, M. J. Fullwood, X. Ruan, Y. Ruan, R. M. Myers, F. Pauli, B. A. Williams, J. Gertz, G. K. Marinov, T. E. Reddy, J. Vielmetter, E. Partridge, D. Trout, K. E. Varley, C. Gasper, A. Bansal, S. Pepke, P. Jain, H. Amrhein, K. M. Bowling, M. Anaya, M. K. Cross, B. King, M. A. Muratet, I. Antoshechkin, K. M. Newberry, K. McCue, A. S. Nesmith, K. I. Fisher-Aylor, B. Pusey, G. DeSalvo, S. L. Parker, S. Balasubramanian, N. S. Davis, S. K. Meadows, T. Eggleston, C. Gunter, J. Newberry, S. E. Levy, D. M. Absher, A. Mortazavi, W. H. Wong, B. Wold, M. J. Blow, A. Visel, L. A. Pennachio, L. Elnitski, E. H. Margulies, S. C. Parker, H. M. Petrykowska, A. Abyzov, B. Aken, D. Barrell, G. Barson, A. Berry, A. Bignell, V. Boychenko, G. Bussotti, J. Chrast, C. Davidson, T. Derrien, G. Despacio-Reyes, M. Diekhans, I. Ezkurdia, A. Frankish, J. Gilbert, J. M. Gonzalez, E. Griffiths, R. Harte, D. A. Hendrix, C. Howald, T. Hunt, I. Jungreis, M. Kay, E. Khurana, F. Kokocinski, J. Leng, M. F. Lin, J. Loveland, Z. Lu, D. Manthravadi, M. Mariotti, J. Mudge, G. Mukherjee, C. Notredame, B. Pei, J. M. Rodriguez, G. Saunders, A. Sboner,

S. Searle, C. Sisu, C. Snow, C. Steward, A. Tanzer, E. Tapanari, M. L. Tress, M. J. van Baren, N. Walters, S. Washietl, L. Wilming, A. Zadissa, Z. Zhang, M. Brent, D. Haussler, M. Kellis, A. Valencia, M. Gerstein, A. Reymond, R. Guig?, J. Harrow, T. J. Hubbard, S. G. Landt, S. Frietze, A. Abyzov, N. Addleman, R. P. Alexander, R. K. Auerbach, S. Balasubramanian, K. Bettinger, N. Bhardwaj, A. P. Boyle, A. R. Cao, P. Cayting, A. Charos, Y. Cheng, C. Cheng, C. Eastman, G. Euskirchen, J. D. Fleming, F. Grubert, L. Habegger, M. Hariharan, A. Harmanci, S. Iyengar, V. X. Jin, K. J. Karczewski, M. Kasowski, P. Lacroute, H. Lam, N. Lamarre-Vincent, J. Leng, J. Lian, M. Lindahl-Allen, R. Min, B. Miotto, H. Monahan, Z. Moqtaderi, X. J. Mu, H. O'Geen, Z. Ouyang, D. Patacsil, B. Pei, D. Raha, L. Ramirez, B. Reed, J. Rozowsky, A. Sboner, M. Shi, C. Sisu, T. Slifer, H. Witt, L. Wu, X. Xu, K. K. Yan, X. Yang, K. Y. Yip, Z. Zhang, K. Struhl, S. M. Weissman, M. Gerstein, P. J. Farnham, M. Snyder, S. A. Tenenbaum, L. O. Penalva, F. Doyle, S. Karmakar, S. G. Landt, R. R. Bhanvadia, A. Choudhury, M. Domanus, L. Ma, J. Moran, D. Patacsil, T. Slifer, A. Victorsen, X. Yang, M. Snyder, T. Auer, L. Centanin, M. Eichenlaub, F. Gruhl, S. Heermann, B. Hoeckendorf, D. Inoue, T. Kellner, S. Kirchmaier, C. Mueller, R. Reinhardt, L. Schertel, S. Schneider, R. Sinn, B. Wittbrodt, J. Wittbrodt, Z. Weng, T. W. Whitfield, J. Wang, P. J. Collins, S. F. Aldred, N. D. Trinklein, E. C. Partridge, R. M. Myers, J. Dekker, G. Jain, B. R. Lajoie, A. Sanyal, G. Balasundaram, D. L. Bates, R. Byron, T. K. Canfield, M. J. Diegel, D. Dunn, A. K. Ebersol, T. Frum, K. Garg, E. Gist, R. Hansen, L. Boatman, E. Haugen, R. Humbert, G. Jain, A. K. Johnson, E. M. Johnson, T. V. Kutyavin, B. R. Lajoie, K. Lee, D. Lotakis, M. T. Maurano, S. J. Neph, F. V. Neri, E. D. Nguyen, H. Qu, A. P. Reynolds, V. Roach, E. Rynes, P. Sabo, M. E. Sanchez, R. S. Sandstrom, A. Sanyal, A. O. Shafer, A. B. Stergachis, S. Thomas, R. E. Thurman, B. Vernot, J. Vierstra, S. Vong, H. Wang, M. A. Weaver, Y. Yan, M. Zhang, J. M. Akey, M. Bender, M. O. Dorschner, M. Groudine, M. J. MacCoss, P. Navas, G. Stamatoyannopoulos, R. Kaul, J. Dekker, J. A. Stamatoyannopoulos, I. Dunham, K. Beal, A. Brazma, P. Flicek, J. Herrero, N. Johnson, D. Keefe, M. Lukk, N. M. Luscombe, D. Sobral, J. M. Vaquerizas, S. P. Wilder, S. Batzoglou, A. Sidow, N. Hussami, S. Kyriazopoulou-Panagiotopoulou, M. W. Libbrecht, M. A. Schaub, A. Kundaje, R. C. Hardison, W. Miller, B. Giardine, R. S. Harris, W. Wu, P. J. Bickel, B. Banfai, N. P. Boley, J. B. Brown, H. Huang, Q. Li, J. J. Li, W. S. Noble, J. A. Bilmes, O. J. Buske, M. M. Hoffman, A. D. Sahu, P. V. Kharchenko, P. J. Park, D. Baker, J. Taylor, Z. Weng, S. Iyer, X. Dong, M. Greven, X. Lin, J. Wang, H. S. Xi, J. Zhuang, M. Gerstein, R. P. Alexander, S. Balasubramanian, C. Cheng, A. Harmanci, L. Lochovsky, R. Min, X. J. Mu, J. Rozowsky, K. K. Yan, K. Y. Yip, and E. Birney, "An integrated encyclopedia of DNA elements in the human genome," *Nature*, vol. 489, pp. 57–74, Sep 2012.

[3] H. Li, "Aligning sequence reads, clone sequences and assembly contigs with bwa-mem," 2013.

[4] A. R. Quinlan, "BEDTools: The Swiss-Army Tool for Genome Feature Analysis," *Curr Protoc Bioinformatics*, vol. 47, pp. 1–34, Sep 2014.

[5] V. Datta, S. Hannenhalli, and R. Siddharthan, "ChIPulate: A comprehensive ChIP-seq simulation pipeline," *PLoS Comput. Biol.*, vol. 15, p. e1006921, 03 2019.

[6] T. Subkhankulova, F. Naumenko, O. E. Tolmachov, and Y. L. Orlov, "Novel ChIP-seq simulating program with superior versatility: isChIP," *Brief Bioinform*, Dec 2020.

[7] R. Thomas, T. S., A. Holloway, and S. Katherine, "Features that define the best ChIP-seq peak calling algorithms," *Brief Bioinform.*, vol. 18, pp. 441–450, May 2017.

[8] P. Humburg, C. A. Helliwell, D. Bulger, and G. Stone, "ChIPseqR: analysis of ChIP-seq experiments," *BMC Bioinformatics*, vol. 12, p. 39, Jan 2011.
